# Semaglutide Versus Empagliflozin in Uncontrolled Type 2 Diabetes: A Cohort Study With 18 Months of Follow-Up (SEMPA18)

**DOI:** 10.7759/cureus.83416

**Published:** 2025-05-03

**Authors:** David Aristizabal-Colorado, David Alexander Vernaza Trujillo, Santiago Sierra Castillo, Wilfredo Antonio Rivera Martinez, Marisol Badiel, Alin Abreu Lomba

**Affiliations:** 1 Interinstitutional Group of Internal Medicine 1 (GIMI1), Universidad Libre, Cali, COL; 2 Epidemiology, Fundación Universitaria del Área Andina, Bogotá, COL; 3 Medicine, Universidad CES, Medellín, COL; 4 Endocrinology, Universidad de Antioquia, Medellín, COL; 5 Internal Medicine, Universidad Libre, Cali, COL; 6 Endocrinology, Imbanaco Clinic, Cali, COL

**Keywords:** empaglifozin, glycemic conrol, renal function, semaglutide, type-2 diabetes mellitus

## Abstract

Introduction: Type 2 diabetes mellitus (T2DM) is a chronic disease that substantially increases morbidity and mortality through its cardiovascular and renal complications. Beyond mere glycemic control, current guidelines from the European Society of Cardiology (ESC) and Kidney Disease: Improving Global Outcomes (KDIGO) recommend interventions that confer additional cardiovascular and renal benefits.

Objective: This study aims to assess and compare the efficacy of semaglutide versus empagliflozin in improving glycemic control, weight, blood pressure, and renal function in patients with uncontrolled T2DM after 18 months of follow-up.

Methods: This retrospective cohort study included 41 patients with uncontrolled T2DM (glycated hemoglobin (HbA1c) > 7%) who received either empagliflozin (n=20) or semaglutide (n=21) as monotherapy. Clinical and laboratory parameters (HbA1c, fasting plasma glucose, body weight, systolic and diastolic blood pressure, serum creatinine, estimated glomerular filtration rate (eGFR), and urine microalbumin) were measured at baseline and 18 months (SEMPA18). Changes were compared between the two groups using appropriate statistical methods.

Results: Of the 41 participants (58.5% male), 20 were treated with empagliflozin and 21 with semaglutide. After 18 months, median HbA1c decreased from 7.60% to 6.85% in the empagliflozin group (100% improved) and from 7.90% to 7.00% in the semaglutide group (95.2% improved). A reduction in body weight of 5% or more was achieved by 2 of 20 (10.0%) empagliflozin-treated patients versus 6 of 21 (28.6%) among those on semaglutide (p=0.48). Albuminuria improved in 18 of 20 (90.0%) empagliflozin users (median final: 12.0 mg/dL) compared to 14 of 21 (66.7%) semaglutide users (median final: 20.0 mg/dL), although the difference was not statistically significant (p=0.07). Both groups showed gains in eGFR (final median: 80.50 vs. 71.00 mL/min/1.73 m²; p=0.048), and serum creatinine decreased in the majority of patients (75.0% vs. 71.4%). A subgroup analysis revealed that heart failure status (LVEF < 50% or documented diagnosis) was associated with less improvement in renal markers, particularly serum creatinine, regardless of the treatment group.

Conclusions: In this real-world, 18-month cohort, empagliflozin produced more pronounced improvements in albuminuria and a slightly greater absolute reduction in HbA1c, whereas semaglutide showed a trend toward greater weight loss, though not statistically significant. These findings emphasize individualized therapy choices in T2DM based on cardiorenal risk profiles and underscore the potential value of early empagliflozin initiation, especially in patients at higher risk of renal deterioration.

## Introduction

Type 2 diabetes mellitus (T2DM) is a major global public health concern due to its increasing prevalence, which is estimated to rise by 10% by 2030, and its strong association with cardiovascular and renal complications, leading to increased morbidity and mortality rates [1]. T2DM management has focused on reducing glycated hemoglobin (HbA1c) levels as a key marker of glycemic control [2]. As new therapies have been developed, it has become evident that glucose control alone is insufficient to address the full spectrum of risks faced by T2DM patients [2]. Cardiovascular complications, including heart disease and stroke, as well as renal complications, play a crucial role in reducing quality of life and increasing mortality in this population [3].

Current guidelines from the European Society of Cardiology (ESC) and Kidney Disease: Improving Global Outcomes (KDIGO) emphasize the importance of therapies that not only ensure adequate glycemic control but also reduce cardiovascular risk and improve renal outcomes. Sodium-glucose cotransporter 2 inhibitors (SGLT2i) and glucagon-like peptide-1 receptor agonists (GLP-1 RAs) have demonstrated efficacy in HbA1c reduction and cardiovascular and renal protection. However, it remains essential to identify new therapeutic agents or conduct comparative evaluations of available medications in specific populations to optimize the early management of T2DM and its complications [4-6].

This real-world study aims to evaluate the impact of early monotherapy with two first-line treatments for T2DM, semaglutide and empagliflozin, by analyzing their efficacy in metabolic control and long-term cardiovascular and renal outcomes (SEMPA18). Identifying differences in effectiveness and safety between these two treatments may provide valuable insights for clinical decision-making, aiding in the personalized management of T2DM patients at high risk for complications.

## Materials and methods

Study design and population

A retrospective cohort study was conducted on patients diagnosed with T2DM in Cali, Colombia, treated at the Endocrinology Unit of the Imbanaco Medical Center between January 2021 and April 2022. Patients initiated treatment with either empagliflozin or semaglutide and were followed for 18 months, with evaluations every six months. Treatment assignment was based on the clinical judgment of the attending physician and institutional protocol.

Patients included in the study were adults aged 18 years or older diagnosed with uncontrolled T2DM (HbA1c > 7%) who were treated with either an SGLT2 inhibitor or a GLP-1 RA and had clinical follow-ups at baseline, 6, 12, and 18 months. Exclusion criteria comprised patients with secondary hypertension, stage 5 chronic kidney disease, those requiring additional antidiabetic medications, pregnant women, and individuals receiving treatment in other institutions.

The study focused on evaluating key clinical parameters, including pre-existing heart failure, left ventricular ejection fraction (LVEF), HbA1c, fasting plasma glucose, systolic and diastolic blood pressure, body weight, albuminuria, and serum creatinine levels. Patients received empagliflozin 25 mg orally once daily or semaglutide 0.5 mg subcutaneously once weekly. As part of the follow-up, each participant underwent a transthoracic echocardiogram to assess cardiac function. LVEF was documented as a key parameter to evaluate ventricular performance and to estimate its potential influence on heart failure risk.

Statistical analysis

The data were collected directly by the medical professional and documented in the medical records of each patient during the follow-up period, following the established protocol and meeting the inclusion and exclusion criteria. Subsequently, the data were tabulated in Excel (Microsoft Corporation, Redmond, USA), where adjustments were made to the database according to the variables defined for the study by the research director. Variables with more than 10% missing data were excluded.

Statistical analyses were performed using IBM SPSS Statistics for Windows, Version 29 (Released 2021; IBM Corp., Armonk, New York, USA). Demographic data were presented as means and standard deviations or as absolute and relative frequencies, depending on the distribution estimated using the Shapiro-Wilk test. Between-group differences were analyzed using Student’s t-test for independent samples, interpreting mean differences, as well as Fisher’s exact test or chi-square tests for categorical variables. For data that did not follow a normal distribution, non-parametric tests, specifically the Mann-Whitney U test, were applied. A 95% confidence interval was established.

## Results

A total of 41 patients with uncontrolled T2DM were included and allocated into two groups: 20 received empagliflozin, and 21 were treated with semaglutide. In the empagliflozin group, 9 out of 20 participants (45.0%) were female, whereas 11 (55.0%) were male; in the semaglutide group, 8 out of 21 (38.1%) were female, and 13 (61.9%) were male. The median age was 59.5 years (interquartile range (IQR): 50.75-68.25) for those on empagliflozin and 60.0 (54.0-66.0) for those on semaglutide. Both groups had a comparable duration of T2DM, with a median of 3.0 years (2.0-6.0 in empagliflozin vs. 2.0-7.0 in semaglutide). Regarding the left ventricular ejection fraction (LVEF), the median was 53.0% (50.0-58.75) in the empagliflozin group and 54.0% (50.0-60.0) in the semaglutide group. Half of the patients in each arm (10 of 20 in empagliflozin and 10 of 21 in semaglutide) had documented heart failure at baseline.

Before starting treatment, the median HbA1c was 7.60% (7.40-8.08) in the empagliflozin group and 7.90% (7.40-8.30) in the semaglutide group, whereas fasting plasma glucose values were 131.0 mg/dL (118.5-150.0) and 117.0 mg/dL (109.0-137.0), respectively. Baseline systolic and diastolic blood pressures were also similar: 128.5 mmHg (120.0-140.75) for systolic in empagliflozin versus 126.0 mmHg (120.0-137.6) in semaglutide, and 84.0 mmHg (78.25-88.0) versus 80.0 mmHg (76.0-86.0) for diastolic. Median body weight was 78.5 kg (69.25-82.0) in the empagliflozin group and 76.0 kg (69.5-85.0) in the semaglutide group.

Initial serum creatinine had a median of 1.06 mg/dL (0.90-1.19) in empagliflozin and 1.00 mg/dL (0.90-1.19) in semaglutide; baseline albuminuria reached 22.50 mg/dL (12.00-55.00) in the first group and 16.00 mg/dL (8.00-27.00) in the second. Finally, the median estimated glomerular filtration rate (eGFR) was 72.0 mL/min/1.73 m² (57.75-95.0) in empagliflozin and 73.0 (65.5-88.0) in semaglutide (Table 1).

**Table 1:** Baseline demographic and clinical characteristics %: percentage; IQR: interquartile range; eGFR: estimated glomerular filtration rate; kg: kilograms; LVEF: left ventricular ejection fraction; n: sample size; HbA1c: glycated hemoglobin; T2DM: type 2 diabetes mellitus; BP: blood pressure

Variable	Empagliflozin (n=20)	Semaglutide (n=21)
Women, n (%)	9 (45.0)	8 (38.1)
Men, n (%)	11 (55.0)	13 (61.9)
Age, years, median (IQR)	59.5 (50.75-68.25)	60.0 (54.0-66.0)
Duration of T2DM, years, median (IQR)	3.0 (2.0-6.0)	3.0 (2.0-7.0)
Heart failure at baseline, n (%)	10 (50.0)	10 (47.6)
LVEF, %, median (IQR)	53.0 (50.0-58.75)	54.0 (50.0-60.0)
HbA1c, %, median (IQR)	7.60 (7.40-8.08)	7.90 (7.40-8.30)
Fasting plasma glucose, mg/dL, median (IQR)	131.0 (118.5-150.0)	117.0 (109.0-137.0)
Systolic BP, mmHg, median (IQR)	128.5 (120.0-140.75)	126.0 (120.0-137.6)
Diastolic BP, mmHg, median (IQR)	84.0 (78.25-88.0)	80.0 (76.0-86.0)
Body weight, kg, median (IQR)	78.5 (69.25-82.0)	76.0 (69.5-85.0)
Serum creatinine, mg/dL, median (IQR)	1.06 (0.90-1.19)	1.00 (0.90-1.19)
Albuminuria, mg/dL, median (IQR)	22.50 (12.00-55.00)	16.00 (8.00-27.00)
eGFR, mL/min/1.73 m², median (IQR)	72.0 (57.75-95.0)	73.0 (65.5-88.0)

After 18 months of follow-up, both groups showed an improvement in glycemic control. The median HbA1c decreased to 6.85% (6.38-7.20) among the 20 empagliflozin-treated patients and to 7.00% (6.70-7.30) in the 21 receiving semaglutide. All individuals in the empagliflozin group (20 of 20, 100%) experienced a reduction in HbA1c, whereas 20 out of 21 (95.2%) in the semaglutide group also achieved lower levels; only one patient (4.8%) failed to improve (p=1.0). Moreover, 14 of 20 (70.0%) on empagliflozin and 12 of 21 (57.1%) on semaglutide attained HbA1c < 7% (p=0.39). With respect to fasting plasma glucose, improvements were observed in 16 of 20 (80.0%) patients on empagliflozin and 18 of 21 (85.7%) on semaglutide, without significant differences.

Body weight decreased in both groups, with a median reduction of 3.93 kg (1.47-4.25) in empagliflozin and 3.00 kg (1.75-4.50) in semaglutide. However, only two of 20 (10.0%) of those on empagliflozin and six of 21 (28.6%) on semaglutide achieved at least a 5% weight loss (p=0.48). Systolic and diastolic blood pressures fell similarly in both arms, without statistically significant differences, reaching values around 120.0 mmHg for systolic and 79-80 mmHg for diastolic at the end of follow-up (Table 2).

**Table 2:** Glycemic and weight outcomes at 18 months %: percentage; IQR: interquartile range; eGFR: estimated glomerular filtration rate; kg: kilograms; LVEF: left ventricular ejection fraction; n: sample size; HbA1c: glycated hemoglobin; FPG: fasting plasma glucose; t-value: t-value for t-Student test; chi-square: chi-square test statistic

Variable	Empagliflozin (n=20)	Semaglutide (n=21)	p-value	t-value	Chi-square
HbA1c, %, median (IQR) at 18 months	6.85 (6.38-7.20)	7.00 (6.70-7.30)	0.03	-2,14	-
Patients with improved HbA1c, n (%)	20 (100)	20 (95.2)	1.00	-0,77	-
Patients with HbA1c <7%, n (%)	14 (70.0)	12 (57.1)	0.39	-	0.73
Fasting plasma glucose, mg/dL, median (IQR) at 18 months	111.50 (100.0-116.25)	114.00 (97.0-130.0)	0.44	1.27	-
Patients with improved FPG, n (%)	16 (80.0)	18 (85.7)	1.00	-	0.23
Body weight change, kg, median (IQR) at 18 months	3.93 (1.47-4.25)	3.00 (1.75-4.50)	0.35	0.45	-
Patients with ≥5% weight loss, n (%)	2 (10.0)	6 (28.6)	0.48	-	2.25

Renal outcomes were also favorable in most patients. Regarding albuminuria, improvement at 18 months was noted in 18 of 20 (90.0%) individuals on empagliflozin, compared with 14 of 21 (66.7%) on semaglutide (p=0.07). The final medians were 12.0 mg/dL (6.0-25.0) and 20.0 mg/dL (8.0-40.0), respectively. A reduction of at least 30% in albuminuria was recorded in 15 of 20 (75.0%) participants in the empagliflozin arm and 15 of 21 (71.4%) on semaglutide, while decreases of ≥50% occurred in 13 of 20 (65.0%) and 10 of 21 (47.6%), respectively.

Improvements in eGFR were seen in 15 of 20 (75.0%) patients on empagliflozin and 15 of 21 (71.4%) on semaglutide (p=0.80), with final values of 80.50 (71.50-95.75) and 71.00 (65.50-80.50), respectively. Regarding serum creatinine, 15 of 20 (75.0%) in empagliflozin and 15 of 21 (71.4%) in semaglutide presented decreased levels, with no significant differences between them (Table 3).

**Table 3:** Renal outcomes at 18 months %: percentage; IQR: interquartile range; eGFR: estimated glomerular filtration rate; t-value: t-value for t-Student test; chi-square: chi-square test statistic

Variable	Empagliflozin (n=20)	Semaglutide (n=21)	p-value	t-value	Chi-square
Albuminuria, mg/dL, median (IQR) at 18 months	12.0 (6.0-25.0)	20.0 (8.0-40.0)	1.00	-2.12	-
Patients with improved albuminuria, n (%)	18 (90.0)	14 (66.7)	0.07	-	3.25
≥30% reduction in albuminuria, n (%)	15 (75.0)	15 (71.4)	–	-	-
≥50% reduction in albuminuria, n (%)	13 (65.0)	10 (47.6)	–	-	-
eGFR, mL/min/1.73 m², median (IQR) at 18 months	80.50 (71.50-95.75)	71.00 (65.50-80.50)	0.48	-0.70	-
Patients with improved eGFR, n (%)	15 (75.0)	15 (71.4)	0.8	-	0.06
Patients with eGFR deterioration, n (%)	5 (25.0)	6 (28.6)	1.00	0.06	-
Serum creatinine, mg/dL, median (IQR) at 18 months	1.00 (0.89-1.10)	1.00 (0.95-1.10)	0.36	-0,684	-
Patients with improved creatinine, n (%)	15 (75.0)	15 (71.4)	0.80	-	0.06
Patients with creatinine deterioration, n (%)	5 (25.0)	6 (28.6)	0.77	-	0.06

A subgroup of 20 patients (10 in each group) with heart failure - defined by an LVEF <50% and/or a documented clinical diagnosis - was compared with 21 patients who did not have this condition. Improvement in HbA1c levels was observed in both heart failure patients and those without this condition. In 100% of patients without heart failure, such improvement was noted, while in the heart failure group, the improvement was 94.1%. However, this difference did not reach statistical significance (p=0.229). Regarding renal outcomes, serum creatinine improved in 21 of 24 individuals (87.5%) without heart failure but in only 9 of 17 (52.9%) among those with this comorbidity (p=0.014), and creatinine deterioration was also higher in patients with heart failure (47.1% vs. 12.5%). Estimated eGFR improved in 20 of 24 (83.3%) participants without heart failure compared with 10 of 17 (58.8%) in those with heart failure, although the difference did not reach statistical significance (p=0.081). Albuminuria showed no significant difference (p=0.185) between both groups, although the proportion of improvement was higher in heart failure patients (88.2% vs. 70.8%) (Table 4).

**Table 4:** Subgroup analysis: patients with and without heart failure %: percentage; chi-square: chi-square test statistic

Outcome	No heart failure (n=24)	Heart failure (n=17)	p-value	Chi-square
Improvement in HbA1c, n (%)	24 (100)	16 (94.1)	0.229	1.447
Improvement in serum creatinine, n (%)	21 (87.5)	9 (52.9)	0.014	6.054
Improvement in eGFR, n (%)	20 (83.3)	10 (58.8)	0.081	3.045
Improvement in albuminuria, n (%)	17 (70.8)	15 (88.2)	0.185	1.759

## Discussion

This study observed that after 18 months of follow-up, the empagliflozin group achieved a 12.5% reduction in total HbA1c (equivalent to a 0.97% absolute decrease), whereas the semaglutide group attained a 10.2% reduction (0.79% absolute decrease). Unlike the findings of large-scale clinical trials, this study suggests better metabolic control with empagliflozin after 18 months of medical therapy. Comparing these outcomes to pivotal trials for each agent, EMPA-REG OUTCOME reported a 0.54% reduction in HbA1c at 12 weeks - albeit with a much shorter follow-up period than either trial to evaluate cardiovascular and other long-term outcomes with semaglutide in subjects with type 2 diabetes (SUSTAIN-6) or the present study [7]. In SUSTAIN 6, semaglutide achieved a mean 1.1-1.4% HbA1c reduction over 104 weeks [8]. The differences between our results and those of pivotal trials may be partly attributed to the study design, as it is an uncontrolled study. Additionally, administrative limitations in medication dispensing could have impacted adherence and, consequently, influenced the final outcomes. Although adverse events were not specifically collected as variables in this study, they may have contributed to the inability to achieve full dosing in some patients, including the occurrence of mild adverse effects such as nausea.

After 18 months, patients in the semaglutide group experienced a greater reduction in body weight, as anticipated. However, they did not reach the levels reported in major trials, and the difference compared to empagliflozin was not statistically significant, even though empagliflozin also promotes weight reduction [3]. In SUSTAIN 6, semaglutide was associated with a weight loss ranging from -3.6 to -4.9 kg, whereas shorter-term studies with empagliflozin have shown reductions of -1.4 to -2.1 kg [8,9]. Our results align with these data, and the lack of statistical significance could stem from the sample size in each group. Notably, 28.6% of 21 patients on semaglutide achieved what we considered a successful weight loss (≥5% of baseline weight), compared to only 10% of those on empagliflozin.

Both groups exhibited a decrease in systolic blood pressure at 18 months, which was slightly greater with semaglutide than empagliflozin. In contrast, the decline in diastolic blood pressure was marginally higher in the empagliflozin group. According to the literature, semaglutide reduces systolic blood pressure by approximately 4-5 mmHg and diastolic blood pressure by 1.5-3.5 mmHg in non-diabetic individuals and around 2 mmHg systolic and 0.1 mmHg diastolic in diabetic patients [10,11]. Empagliflozin, on the other hand, has been associated with systolic blood pressure reductions of around 9 to 12 mmHg - particularly in patients with a lower body mass index - and diastolic blood pressure reductions of 2 to 8 mmHg [12,13]. The diastolic blood pressure drop observed here is consistent with the diuretic and hemodynamic optimization effects characteristic of SGLT2 inhibitors [14].

Albuminuria reached significantly lower levels in the empagliflozin group, showing a statistically significant reduction compared to the semaglutide group, in which proteinuria tended to increase slightly. This aligns with the well-documented nephroprotective properties of SGLT2 inhibitors, attributed to both hemodynamic and anti-inflammatory effects [3]. We consider this the most important finding of our study, as it supports favoring early SGLT2 inhibitor therapy in patients with T2DM whose risk of renal deterioration may outweigh other treatment priorities, such as weight loss. These results corroborate the findings of Simms-Williams et al. and Riley et al., whose real-world cohort studies reported no significant difference in renal outcomes when comparing SGLT2 inhibitors plus GLP-1 RAs versus SGLT2 inhibitors alone. However, in contrast, patients who received a GLP-1 RA plus an SGLT2 inhibitor experienced a positive reduction in renal events [15,16].

In the present study, both groups showed improved eGFR, although it was slightly better with semaglutide, with no statistically significant difference. Upon evaluating the renal impact of these drugs, we observed that a greater decline in eGFR was more common in patients with heart failure than in those without (41.2% vs. 16.7%). Among patients without heart failure (or reduced ejection fraction), there was a statistically significant improvement in serum creatinine in the empagliflozin group compared with semaglutide at 18 months of follow-up.

To date, there are no randomized clinical trials that directly compare subcutaneous semaglutide with empagliflozin. However, indirect evaluations have suggested that semaglutide may provide greater weight loss along with better HbA1c control, findings that were not definitively borne out in our study. Although semaglutide did tend to yield better weight reduction, the difference was not statistically significant [17]. Rodbard et al. conducted a comparison between oral semaglutide and empagliflozin, showing no significant difference in weight loss at 26 weeks but demonstrating semaglutide superiority at 52 weeks; semaglutide also produced better HbA1c control throughout follow-up [2]. Furthermore, cost-effectiveness analyses suggest that empagliflozin may be more cost-effective in preventing cardiovascular events when compared to semaglutide [18].

Overall, this study partially supports the notion that patients at high cardiorenal risk may benefit from the early initiation of empagliflozin, whereas patients with obesity and a lower cardiorenal risk might be better served by semaglutide for weight management and prevention of cerebrovascular disease. In cases of markedly elevated HbA1c, combined therapy with these two agents may be the most logical strategy [19]. Our findings also indicate that for individuals at high risk of chronic kidney disease progression, early use of an SGLT2 inhibitor can be particularly advantageous, especially before left ventricular ejection fraction shows signs of significant reduction.

Strengths

This is a real-world study with a retrospective 18-month follow-up, providing an extended assessment of treatment effects in clinical practice. Monotherapy was maintained in each group for a prolonged period, reducing variability due to concomitant antidiabetic medications. A detailed analysis of metabolic, cardiovascular, and renal parameters was included, allowing a comprehensive evaluation of empagliflozin and semaglutide effects. The study design enables the assessment of glycemic control, renal function, and blood pressure in a real-world clinical setting, offering valuable insights into the effectiveness of these treatments in uncontrolled T2DM patients.

Limitations

The small sample size limits the generalizability of the findings and the ability to detect significant differences. Larger trials could provide more robust evidence and improve the ability to identify meaningful differences. Retrospective design may introduce data collection biases and limit causal inferences. There was a lack of randomization, potentially leading to baseline differences between groups that may affect the interpretation of results. Clinical follow-up every six months may fail to capture short-term fluctuations in the assessed parameters. Strict exclusion criteria could limit the applicability of findings to more diverse populations of T2DM patients.

## Conclusions

In patients with type 2 diabetes, empagliflozin demonstrated a beneficial effect on albuminuria and HbA1c, as well as a greater decrease in diastolic blood pressure compared with semaglutide over 18 months of follow-up. Semaglutide, in turn, showed a more pronounced weight reduction and a greater decrease in systolic blood pressure. Serum creatinine improved more notably in those with a physiologically preserved ejection fraction. These findings support the early use of empagliflozin in patients with type 2 diabetes and heart failure to promote renal protection via SGLT2 inhibitors.
